# Topical nerve growth factor prevents neurodegenerative and vascular stages of diabetic retinopathy

**DOI:** 10.3389/fphar.2022.1015522

**Published:** 2022-09-12

**Authors:** Gianpaolo Zerbini, Silvia Maestroni, Letizia Leocani, Andrea Mosca, Michela Godi, Renata Paleari, Arianna Belvedere, Daniela Gabellini, Paola Tirassa, Valerio Castoldi, Ilaria Viganò, Silvia Galbiati, Valentina Turco, Alessandro Lambiase, Paolo Rama

**Affiliations:** ^1^ Complications of Diabetes Unit, Diabetes Research Institute, IRCCS Ospedale San Raffaele, Milan, Italy; ^2^ Experimental Neurophysiology Unit, INSPE-Institute of Experimental Neurology, IRCCS Ospedale San Raffaele, Milan, Italy; ^3^ Vita-Salute San Raffaele University, Milan, Italy; ^4^ Dipartimento di Fisiopatologia Medico-Chirurgica e dei Trapianti and Centro per la Riferibilità Metrologica in Medicina di Laboratorio (CIRME), Università degli Studi di Milano, Milano, Italy; ^5^ Istituto di Tecnologie Biomediche, Consiglio Nazionale delle Ricerche (ITB-CNR), Milano, Italy; ^6^ National Research Council (CNR), Institute of Biochemistry & Cell Biology (IBBC), Rome, Italy; ^7^ Department of Sense Organs, “Sapienza” University of Rome, Rome, Italy; ^8^ Cornea and Ocular Surface Unit, IRCCS Ospedale San Raffaele, Milan, Italy

**Keywords:** diabetic retinopathy, nerve growth factor, prevention, neurodegeneration, topical treatment

## Abstract

Specific and effective preventive treatment for diabetic retinopathy (DR) is presently unavailable, mostly because the early stages of the complication have been, until recently, poorly understood. The recent demonstration that the vascular phase of DR is preceded and possibly caused by the neurodegeneration of retinal ganglion cells suggests that DR could, at least theoretically, be prevented through an early neuroprotective approach. The aims of our study were to clarify the natural history of diabetes-driven retinal neurodegeneration and to verify the possibility to prevent DR using topical nerve growth factor (NGF). The results of the study show that retinal neurodegeneration, characterized by the loss of retinal ganglion cells represents a relatively early phenomenon of diabetes (between 5 and 16 weeks of age), which tends to be self-limiting in the long run. Neurodegeneration is followed by the development of DR-related vascular dysfunctions, as confirmed by the development of acellular capillaries and the loss of retinal pericytes. Both retinal neurodegeneration and subsequent vascular dysfunction can be successfully prevented by topical NGF administration. These findings suggest that: 1) The first stage of DR consists in a self-limiting retinal neurodegeneration 2) The demonstrated effectiveness of topical NGF in the prevention of DR could be rapidly translated into clinical practice.

## Introduction

It takes several years to move from the onset of diabetes (both type-1 and type-2) to the development of retinal microaneurysms, the first clinical sign of diabetic retinopathy (DR) (Antonetti et al., 2012). Once these abnormalities have appeared, controlling their evolution becomes difficult and only laser photocoagulation and/or intravitreal anti-VEGF treatment are effective during the final stage of the complication (Antonetti et al., 2012; Ting and Wong, 2017).

It is reasonable to assume that the “silent” interval, spanning between onset of diabetes and development of microaneurysms, might represent the ideal time window to start a successful strategy to prevent DR (Lorenzi, 2006). Attempts carried out to reach this aim have been so far unsuccessful mainly because, until recently, the initial dysfunctional mechanisms leading to the development of DR have been poorly understood.

A number of studies over the last few years (Lieth et al., 2000; Fletcher et al., 2005; Antonetti et al., 2006; Santos et al., 2017) have suggested that the vascular phase of DR could be preceded by a diabetes-driven neurodegenerative process affecting, in particular, the retinal ganglion cells (RGC). Natural history and clinical relevance of this phenomenon remain however unknown and whether early neurodegeneration may or may not represent an indispensable and pharmacologically targetable step in the pathogenesis of DR is still under debate (Hammes et al., 1995; Simó et al., 2018).

To clarify these issues, we: 1) investigated and characterized in a mouse model of spontaneous diabetes (Ins2akita) (Barber et al., 2005) the morphological and functional evolution of hyperglycemia-driven retinal neurodegeneration;

2) verified the possibility to prevent both retinal neurodegeneration and subsequent vascular phase of DR through early treatment with recombinant human nerve growth factor (rhNGF, here called NGF) eye drops (topically applied NGF has been shown to reach retina, optic nerve and brain in rodents (Lambiase et al., 2005; Lambiase et al., 2007) and to specifically protect RGC (Guo et al., 2020)).

## Materials and methods

The study was approved by the Institutional Animal Care and Use Committee (IACUC) of the San Raffaele Scientific Institute in Milan, in accordance with National Legislation (D.L. 116/1992) and the European Directive (2010/63/EU) concerning the use of laboratory animals, and with the license of the Italian Board of Health.

### Design of the study

**FIGURE 1:**
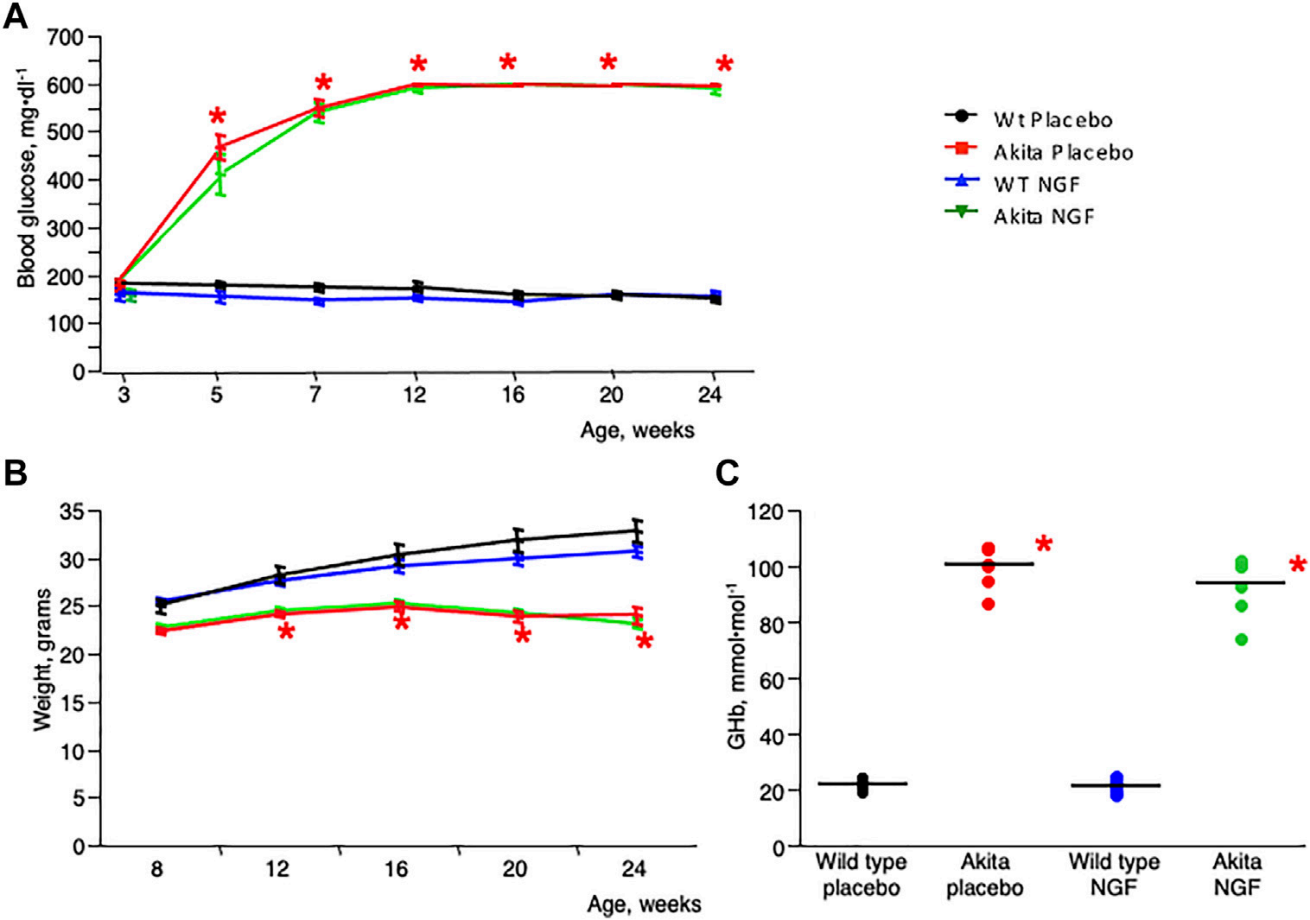
Clinical characteristics. **(A)**. Change of blood glucose levels with time in the four animal groups included in the study. Blood glucose level was similar in the four groups at the beginning of the study (3 weeks of age). In the subsequent time points the two akita groups showed a significantly increased blood glucose level when compared to the two wild type groups. *p < 0.05. **(B)**. Change of body weight with time in the four groups of animals included in the study. The two akita groups show a significantly decreased body weight when compared to the two wild type groups. *p < 0.05. **(C)**. Glycated hemoglobin (GHb) levels at the end of the study (24 weeks) were significantly higher in the akita mice (independent of treatment) compared to wild type animals *p < 0.05.

Four groups of seven animals were studied for 21 weeks (between 3 and 24 weeks of age). At the beginning of the study (3 weeks of age), blood glucose levels of all the animals (both wild type and akita) were similar (as shown in Figure 1A). The animals were therefore randomly assigned to the treatment with placebo (vehicle) or NGF. The groups were topically treated (two drops per eye per day starting at 3 weeks of age) either with placebo (vehicle) or with NGF (180 mg/ml) as follows: 1) Placebo-treated wild type (C57BL/6J) mice; 2) Placebo-treated akita mice; 3) NGF-treated wild type mice; 4) NGF-treated akita mice. Eye drops recombinant human NGF (rhNGF, here called NGF) was provided by Dompé S.p.A. L’Aquila, Italy. Only male animals were studied, as akita females show significantly lower blood glucose levels (Barber et al., 2005).

Thickness of retinal neuron layers was sequentially (3, 5, 8, 16, and 24 weeks of age) evaluated by optical coherence tomography (OCT). Neuroretinal function was evaluated (8, 16, and 24 weeks of age) by electroretinogram (ERG). The animals were sacrificed at the end of the study. The number of RGC was evaluated in the left eye after Brn3a staining (Nadal-Nicolás et al., 2009) (goat polyclonal anti-Brn3a, Santa Cruz, Santa Cruz, CA, United States) while the number of acellular capillaries and pericytes was evaluated in the right eye after performing a trypsin digest (Dietrich and Hammes, 2012). An extra set of seven animals per group was included, treated as previously described and sacrificed at 8 weeks of age to evaluate the number of RGC.

### Optical coherence tomography

*In vivo* analysis of the retina was carried out using Micron IV together with Image-Guided 830 nm OCT (Phoenix Research Laboratories, Pleasanton, CA, United States). The animals were anesthetized with intraperitoneal injection of 80 mg/kg Ketamine, 10 mg/kg Xylazine (Sigma-Aldrich, Munich, Germany). OCT scans were acquired in mydriatic animals through a bidimensional scan (B-scan), performing a 550 μm diameter circular scan around the optic nerve head. Both eyes were examined and results were averaged. Retinal layer segmentation and quantification was performed using Insight software (Phoenix Research Laboratories). Results shown here concern the sum of the thicknesses of Retinal Nerve Fiber Layer (RNFL, that contains RGC axons) plus Ganglion Cell Layer (GCL, that contains RGC bodies). The measurement of RNFL/GCL complex in the mouse is preferable to the measurement of RNFL alone that is usually too thin to be correctly quantified (Jagodzinska et al., 2017).

### Electroretinogram

Mice were dark-adapted for 2 h before the recordings and all procedures were conducted under dim-red light (Pinto et al., 2004). Briefly, mice were anesthetized as above. Body temperature was maintained with a homeothermic pad at 37°C (Harvard Apparatus, Holliston MA, United States). ERG was concurrently recorded from left and right mydriatic eyes using two corneal ERG electrodes connected to a Micromed amplifier (SystemPlus Evolution-Micromed s.p.a., Mogliano Veneto, Italy). Data were acquired at a sampling frequency of 4096 Hz, coded with 16 bits and filtered between 5–70 Hz. Flash stimuli, with intensity of 231 mJ and duration of 10 µs, were delivered to both eyes at a frequency of 0.5 Hz with a Flash10s photo stimulator (Micromed) (Giannelli et al., 2018). For each session, six series (3 for each eye) of 10 flash stimuli were mediated and used for measuring the amplitude of a-wave (baseline to negative a-wave peak) and b-wave (negative a-wave peak to positive b-wave peak).

### Trypsin digest

Trypsin digest was performed as described by Dietrich and Hammes (2012). After hematoxylin and eosin staining the slides were scanned via Aperio^®^ ePathology digital scanner and images were analyzed with ImageScope™ software (both from Leica Biosystems, Nussloch, Germany). The total number of acellular capillaries and pericytes was counted in ten randomly chosen fields for each retina and corrected for the capillary density (number of acellular capillaries/pericytes per mm2 of capillary area).

### Glycated hemoglobin

For the quantification of glycated hemoglobin (GHb), an automated HPLC analyzer, based on boronate affinity chromatography, was used (Premier Hb9210, Trinity Biotech, Menarini, Firenze, IT, United States).

Blood samples were studied as haemolysates in specific racks. An internal quality control process was performed per each analytical run by assaying two control materials with low and high HbA1c level, supplied by the manufacturer.

### Statistics

Data are shown as arithmetical means ± SE. Comparisons between groups were addressed by ANOVA, and multiple comparisons were performed with the Tukey-Kramer test (JMP software for the Apple Macintosh; SAS Institute, Cary, NC). The null hypothesis was rejected at the 5% level (two tailed).

## Results

During the study, blood glucose levels became rapidly and significantly higher in akita groups (Figure 1A) compared to controls. Weight was progressively lower in akita mice (Figure 1B), as expected in animals with heavy glycosuria. Hyperglycemic stability in akita groups was confirmed after measurement of glycated hemoglobin (GHb) at the end of the study (Figure 1C). In particular GHb concentrations were significantly higher in placebo-treated akita mice (100.4 ± 2.5 mmol/mol, mean ± SE) and in NGF-treated akita mice (93.7 ± 2.5) when compared to placebo-treated wild type mice (21.8 ± 2.4) and to NGF-treated wild type mice (21.2 ± 2.5).

**FIGURE 2:**
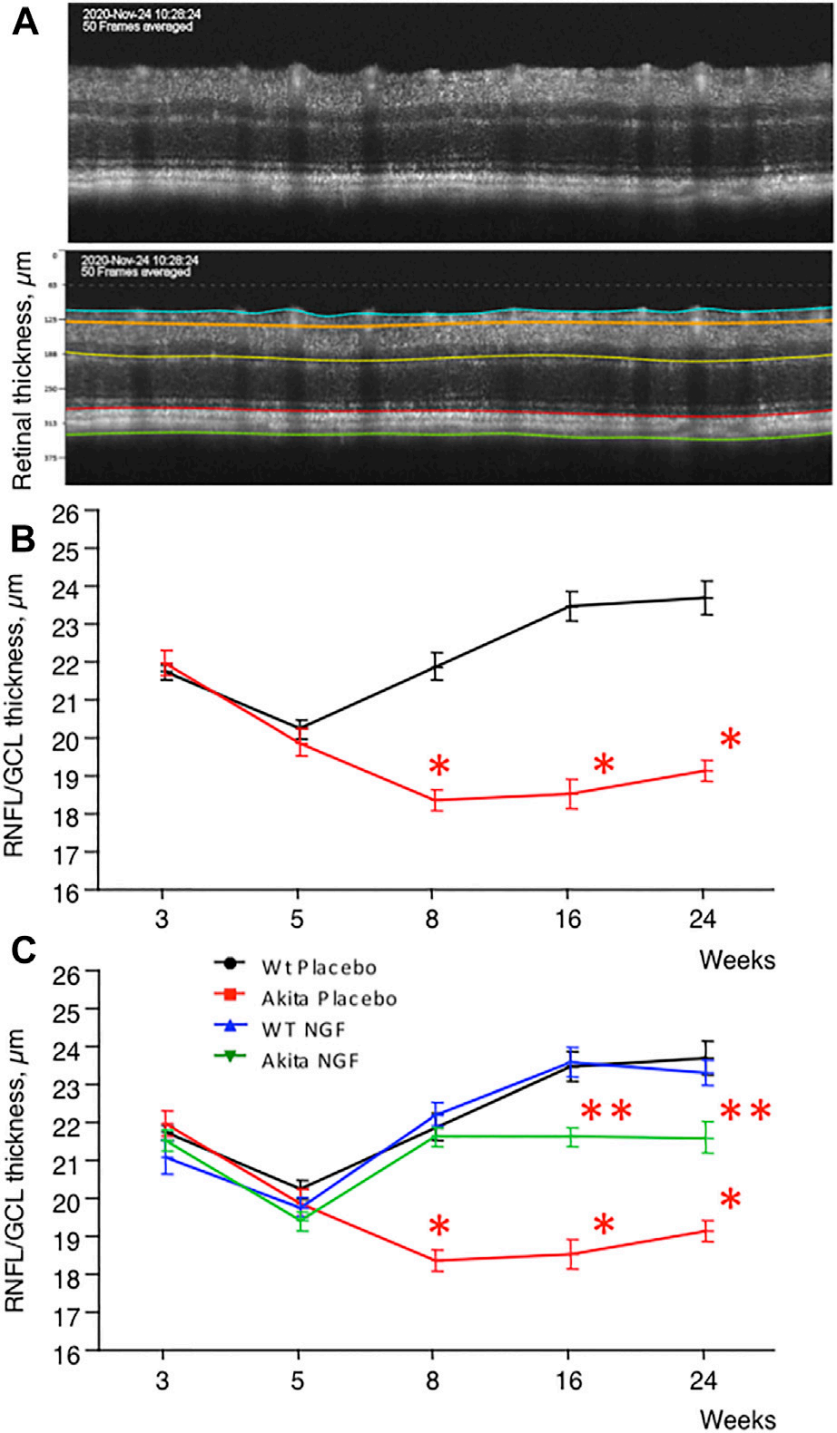
RNFL/GCL complex, time course. **(A)**. Upper panel: retina of a control (C57BL6J) mouse as seen by OCT. Lower panel: Segmentation of the retina. The upper layer is the one specifically considered in this study (RNFL/GCL complex). **(B)**. Time course (3–24 weeks) measurement of RNFL/GCL thickness in placebo-treated akita mice (red line) compared to placebo-treated wild type mice (black line). Placebo-treated akita mice shows significant thickness reduction (*p < 0.05) at 8, 16, and 24 weeks of age. **(C)**. RNFL/GCL thickness increased significantly (*p < 0.05) at 8, 16, and 24 weeks of age in NGF-treated akita mice (green line) when compared to placebo-treated akita mice (red line) even though it remained significantly thinner (**p < 0.05) than in placebo-treated (black line) and NGF-treated (blue line) wild type mice.

As shown in Figures 2A,B, the RNFL/GCL complex, became progressively thinner in akita mice compared to control animals during the first weeks of diabetes and then substantially stabilized after the eighth week of age. Topical treatment with NGF resulted in a significant maintenance of RNFL/GCL thickness in akita mice (green line, Figure 2C). NGF treatment of control animals did not affect RNFL/GCL thickness (blue line, Figure 2C).

**FIGURE 3:**
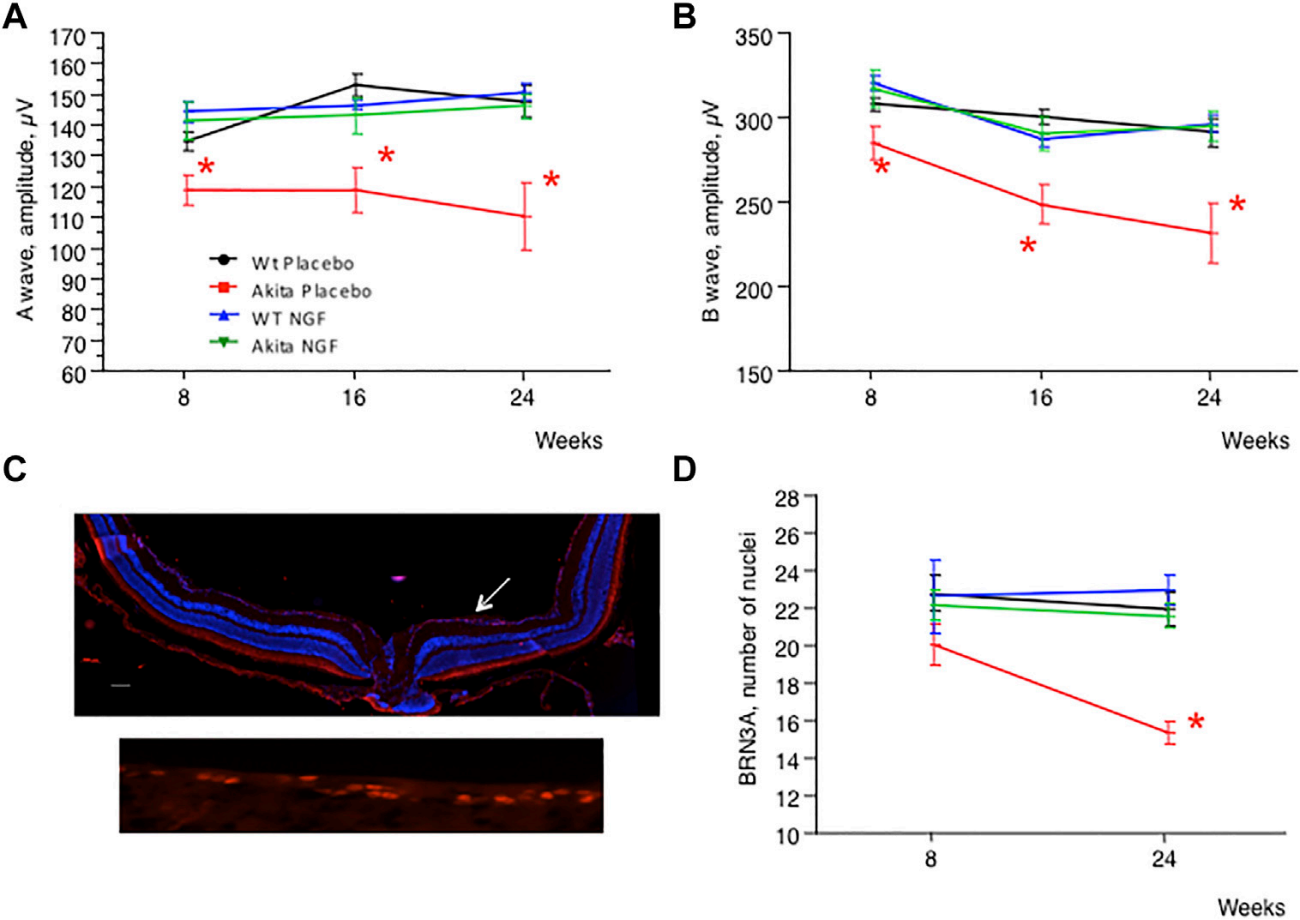
ERG and number of RGC cells, time course. **(A)**. Time course (8–16-24 weeks) measurement of ERG A wave amplitude in the four animal groups considered. The placebo-treated akita group (red line) shows significant amplitude reduction (*p < 0.05) at 8, 16, and 24 weeks of age when compared to placebo-treated wild type mice (black line). The dysfunction improves significantly (*p < 0.05) when akita mice are treated with NGF (green line) and there are no differences between NGF-treated akita mice, placebo-treated wild type mice and NGF-treated wild type mice (blue line). **(B)**. Time course (8–16-24 weeks) measurement of ERG B wave amplitude in the four animal groups considered. The placebo-treated akita group (red line) shows significant amplitude reduction (*p < 0.05) at 8, 16 and 24 weeks of age when compared to placebo-treated wild type mice (black line). The dysfunction improves significantly (*p < 0.05) when akita mice are treated with NGF (green line) and there are no differences between NGF-treated akita mice, placebo-treated wild type mice and NGF-treated wild type mice (blue line). **(C)**. Upper panel: immunofluorescence for the RGC nuclear antigen Brn3a in a retinal section of a control (C57BL/6J) mouse. The arrow indicates the layer formed by the nuclei of RGC (red staining). Lower panel: detail of the nuclei of RGC stained for Brn3a. **(D)**. Time course (8–24 weeks) measurement of the number of RGC cells in the four animal groups considered. No difference between the groups could be demonstrated at 8 weeks of age. The placebo-treated akita group (red line) shows significant numerical reduction (*p < 0.05) at 24 weeks of age when compared to the other three groups. This dysfunction improves significantly when akita mice are treated with NGF (green line) to the point that there are no differences between NGF-treated akita mice, placebo-treated wild type mice (black line) and NGF-treated wild type mice (blue line).

To clarify whether RNFL/GCL thickness reduction was paralleled by functional abnormalities, ERG was performed on each animal involved in the study. As shown in Figures 3A,B, placebo-treated akita mice were characterized by reduced activity of both A and B waves, a dysfunction that was substantially prevented by NGF treatment. No effect on ERG came from NGF treatment of control animals.

Final confirmation that RNFL/GCL thickness reduction and ERG abnormalities were the consequence of RGC loss was obtained by counting RGC in the retina of sacrificed animals after staining for Brn3a (Figure 3C), a specific nuclear antigen of RGC (Nadal-Nicolás et al., 2009). As shown in Figure 3D, at 8 weeks of age RGC count was similar in all the groups considered, but at 24 weeks of age RGC count was significantly lower in placebo-treated akita mice compared to controls. NGF treatment fully prevented RGC loss in akita mice and, once again, NGF had no effect on RGC count in control animals.

**FIGURE 4:**
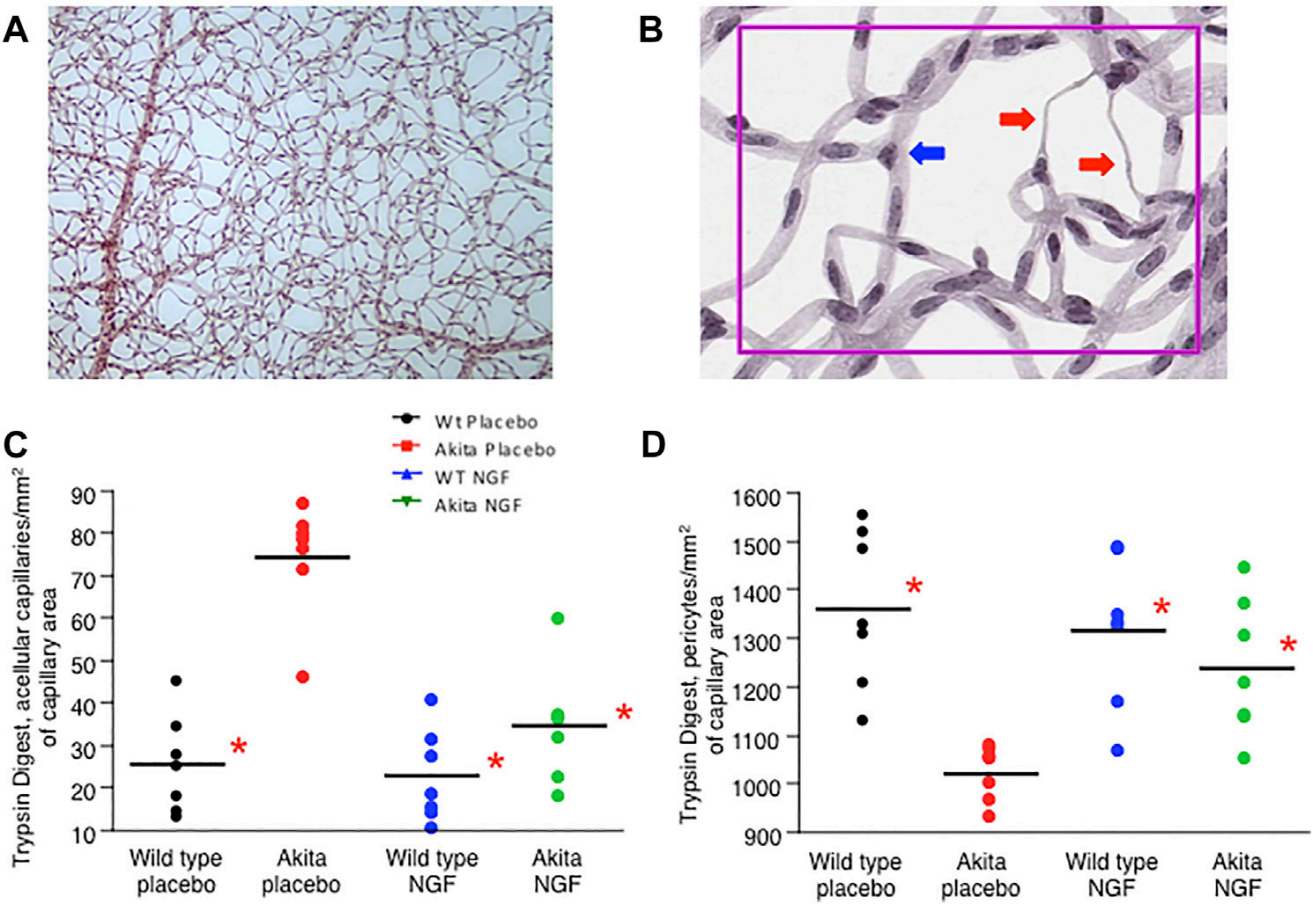
Trypsin digest. **(A)**. Trypsin digestion of a murine retina. **(B)**. Details of trypsin digestion of a murine retina. The presence of acellular capillaries can be appreciated (red arrows). Triangular nuclei are the hallmark of pericytes (blue arrow). **(C)**. The number of retinal acellular capillaries at the end of the study (24 weeks) was significantly higher (*p < 0.05) in the placebo-treated akita mice (red dots) when compared to placebo-treated wild type mice (black dots), to NGF-treated wild type mice (blue dots) and to NGF-treated akita mice (green dots). The last three groups were similar to each other (P=NS) confirming that NGF treatment normalizes the number of retinal acellular capillaries in akita mice. **(D)**. The number of retinal pericytes at the end of the study (24 weeks) was significantly lower (*p < 0.05) in the placebo-treated akita mice (red dots) when compared to placebo-treated wild type mice (black dots), to NGF-treated wild type mice (blue dots) and to NGF-treated akita mice (green dots). The last three groups were similar to each other (P=NS) confirming that NGF treatment normalizes the number of retinal pericytes in akita mice.

To clarify whether early retinal neurodegeneration plays a relevant role in the pathogenesis of DR and whether NGF treatment can also prevent the vascular stage of DR, trypsin digestion was carried out in the retinas of sacrificed animals to search for acellular capillaries and pericyte dropouts (Figures 4A,B), two features of the vascular phase of DR that are shared by humans and mice (Hammes et al., 1995). As shown in Figure 4C, the number of acellular capillaries was significantly higher in placebo-treated akita mice (74.3 ± 6.5 number/mm^2^ of capillary area, mean ± SE) when compared to placebo-treated wild type mice (25.4 ± 4.4) and to NGF-treated wild type mice (22.5 ± 4.0). NGF treatment resulted in a significant reduction and consequent “normalization” of acellular capillaries number in akita mice (34.7 ± 5.5).

In parallel (Figure 4D), the number of retinal pericytes was significantly reduced in placebo-treated akita mice (1021.8 ± 38.1 number/mm^2^ of capillary area, mean ± SE) when compared to placebo-treated wild type mice (1360.3 ± 55.4) and to NGF-treated wild type mice (1316.0 ± 54.0). As above, NGF treatment substantially “normalized” the number of pericytes in akita mice (1237.4 ± 57.5).

## Discussion

The results of the study demonstrate that the natural history of diabetes-driven retinal neurodegeneration, as characterized by OCT (Figure 2B), consists of a relatively early (between 5 and 16 weeks of age), self-limiting phenomenon that shows no tendency to worsen in the final period of observation (between 16 and 24 weeks of age). Only approximately 20–25% of RGC were actually lost during the entire 21-weeks period of observation.

Another important point is that topical NGF treatment is able not only to prevent neurodegeneration, as confirmed from morphologic (OCT), functional (ERG) and histologic (Brn3a) analyses, but also to avoid the development of the vascular stage of DR, which indicates at the same time neurodegeneration as an essential step in the pathogenesis of DR and topical NGF as an effective preventive treatment.

The discovery that, in our mouse model of diabetes, retinal neurodegeneration develops early and tends not to progress with time reproduces and reasonably explains the clinical finding that, even though neurodegeneration represents an early retinal dysfunction (Simó and Hernández, 2012) in at least a subset of diabetic patients (Santos et al., 2017), there is no indication that the dysfunction may worsen with time (Sacconi et al., 2020).

A major question on this regard concerns the reason why, as described in Figure 2B, the diabetes-driven neurodegenerative process tends to stabilize with time while, as shown in Figure 1A, hyperglycemia is not treated or corrected in any way. One possibility is linked to the evidence that RGC are not a homogeneous cellular population as at least 40 subtypes have been identified (Laboissonniere et al., 2019). Although the different subsets have not yet been characterized, a different response to glucose toxicity in different type of cells cannot be excluded, in particular when considering that retinal neurons, at difference with the ones of the brain, rely mostly on aerobic glycolysis (Hurley et al., 2015), thus possibly justifying the hypothesis that a subgroup of glucose “resistant” RGC may survive the death of the “sensitive” ones.

Another possible and in some way fascinating hypothesis is that RGC might progressively become tolerant to hyperglycemia, something similar to what happen to endothelial cells chronically exposed to high ambient glucose (de Zeeuw et al., 2015).

This is not the first time that topical administration of neuroprotective agents such as GLP-1 Receptor Agonists (Hernández et al., 2017), DPP-IV inhibitors (Hernández et al., 2016) and dual endothelin receptor antagonist Bosentan (Bogdanov et al., 2018) were shown to be useful in experimental models of diabetic retinopathy DR. NGF in particular was found to be protective after both systemic (Hammes et al., 1995) and topical administration (Mantelli et al., 2014). If topical administration of NGF will be confirmed to be effective also on the human retina, as suggested by a first study (Lambiase et al., 2009), prevention of DR could become a feasible and realistic task also when the treatment is started at a very young age.

The finding that RNFL-GCL between 3–5 weeks of age tends to become thinner in all the groups of animals considered in the study (Figure 2B) is for sure intriguing but, at the very end, not so surprising. A previous study (Brais-Brunet et al., 2021) in control mice shows that, when measured consecutively by OCT between postnatal days 7 and 21, the thicknesses of four retinal layers (RNFL, IPL, INL and ORL) are progressively and significantly changing, suggesting a temporal “plasticity” of the neural retina. Unfortunately, the above-described study was stopped at 21 days of age and cannot therefore be of use to quantify the change of RNFL thickness during the period of interest (3–5 weeks of age).

Finally, our results also show that in diabetic animals the thinning of RNFL/GCL complex precedes and predicts the loss of RGC (at 8 weeks of age thinning of RNFL/GCL is already significant in placebo-treated akita mice (Figure 2B) while the number of RGC is still similar among the groups considered (Figure 3D), hence qualifying as a new biomarker for both neurodegenerative and vascular stages of DR.

Limitations of the study: 1) To monitor the development of the vascular phase of diabetic retinopathy we counted pericytes + acellular capillaries after the demonstration that trypsin digest presently represents the gold standard method to analyze the retinal vasculature (Chou et al., 2013) and because this technique has been previously used to identify retinal dysfunctions common to humans and animal models (Mizutani et al., 1996). Evaluation of vascular leakage by means of the Evans blue method (Xu et al., 2001), or staining of the retina looking for markers specific for endothelial cells or pericytes would have for sure added important functional and morphological results but could not be done in our case because of the limited number of animals (and consequently of retinas) included in the study. 2) Diabetes-driven changes of RNFL/GCL and microvasculature cannot be directly compared in our study because pericytes and acellular capillaries were quantified only at the end (24 weeks) of the study. A time course study aimed to clarify the natural histories of retinal neurodegeneration and microvascular dysfunction induced by diabetes would have been very interesting but unfortunately it goes beyond the aims of the present study.

In conclusion this study shows, in an animal model of diabetes, that DR is characterized by two consecutive stages (neurodegenerative and vascular) and that, by preventing neurodegeneration through NGF topical treatment, it is possible to avoid the development of the microvascular one, known to be particularly aggressive in humans.

## Data Availability

The raw data supporting the conclusions of this article will be made available by the authors, without undue reservation.
